# Supplementary material for: End Sequence Analysis Toolkit (ESAT) expands the extractable information from single-cell RNA-seq data
Source: Genome Res. 2016 Oct;26(10):1397–410. doi: 10.1101/gr.207902.116 (PMC5052061; doi:10.1101/gr.207902.116)
Supplement: Supplemental Material [file supp_gr.207902.116_Supp_Materials.tar.gz › ESAT-esat_v0.1/doc/html/classjaligner_1_1_sequence-members.html]

ESAT: Member List


ESAT


- Main Page
- Related Pages
- Packages
- Classes
- Files

- Class List
- Class Index
- Class Hierarchy
- Class Members

All Classes Namespaces Files Functions Variables Enumerator Pages

- jaligner
- Sequence

jaligner.Sequence Member List

This is the complete list of members for jaligner.Sequence, including all inherited members.

|  |  |  |
| --- | --- | --- |
| acidAt(int index) | jaligner.Sequence |  |
| getDescription() | jaligner.Sequence |  |
| getId() | jaligner.Sequence |  |
| getSequence() | jaligner.Sequence |  |
| getType() | jaligner.Sequence |  |
| length() | jaligner.Sequence |  |
| NUCLEIC | jaligner.Sequence | static |
| PROTEIN | jaligner.Sequence | static |
| Sequence() | jaligner.Sequence |  |
| Sequence(String sequence) | jaligner.Sequence |  |
| Sequence(String name, char[] sequence) | jaligner.Sequence |  |
| Sequence(String name, String sequence) | jaligner.Sequence |  |
| Sequence(String sequence, String id, String description, int type) | jaligner.Sequence |  |
| setDescription(String description) | jaligner.Sequence |  |
| setId(String id) | jaligner.Sequence |  |
| setSequence(String sequence) | jaligner.Sequence |  |
| setType(int type) | jaligner.Sequence |  |
| subsequence(int index, int length) | jaligner.Sequence |  |
| toArray() | jaligner.Sequence |  |
| toString() | jaligner.Sequence |  |


---

Generated on Fri Aug 15 2014 15:11:05 for ESAT by  

 1.8.7
